# Flow cytometry detection of sustained humoral immune response (IgG + IgA) against native spike glycoprotein in asymptomatic/mild SARS-CoV-2 infection

**DOI:** 10.1038/s41598-021-90054-4

**Published:** 2021-05-21

**Authors:** Paula Piñero, Francisco M Marco De La Calle, Lydia Horndler, Balbino Alarcón, Marisol Uribe Barrientos, Héctor Sarmiento, Fabián Tarín

**Affiliations:** 1 ISABIAL, Hospital General de Alicante, Alicante, Spain; 2 Hospital General de Alicante, Alicante, Spain; 3 Centro de Biología Molecular Severo Ochoa, Madrid, Spain; 4 Hospital General de Valencia, Valencia, Spain

**Keywords:** Biological techniques, Immunology

## Abstract

SARS-CoV-2 is the virus that causes the disease called COVID-19, which has caused the worst pandemic of the century. Both, to know the immunological status of general population and to evaluate the efficacy of the vaccination process that is taking place around the world, serological tests represent a key tool. Classic serological tests, based on colorimetric techniques, such as ELISA or CLIA, continue to be the most widely used option. However, a real improvement in results is still needed. We developed a highly sensitive and specific FCM assay that allows the detection of IgG and IgA antibodies, directed against the native and functional S-protein of SARS-CoV-2 exposed on the membrane of a transfected cell line, up to 8 months after infection.

SARS-CoV-2 is a Baltimore class IV RNA virus that spreads easily through the respiratory tract and causes an infectious disease called COVID-19^1,2^. Since its detection in Wuhan in December 2019, COVID-19 has evolved into a pandemic, causing a global health emergency^3^.

SARS-CoV-2 infection promotes the generation of T and B cell responses, particularly against the spike glycoprotein (S-protein) blocking its ability to bind to ACE-2 protein^4-6^. However, it is still unknown whether the immune response leads to a prolonged protection against reinfection^6–8^.

The evaluation of the immune status against SARS-CoV-2 in the population is of vital importance to know the actual proportion of people who have recovered from the disease, as well as to detect individuals with active infections^9^. Different serological approaches to detect specific antibodies against SARS-CoV-2 S-protein (anti-SCoV2) and the functional activity directed to the formation of S-protein/ACE-2 complexes have been successfully used^10–12^. The Enzyme-Linked ImmunoSorbent Assay (ELISA) and chemiluminescence immunoassay (CLIA) have shown to detect seropositivity in the general population reaching good sensitivity and specificity^10–12^. However, it has been noted the need for improved specificity and sensitivity. In fact, several groups have developed refined techniques previously used with other coronaviruses^13^.

Recently, some publications highlighted the promising role of flow cytometry (FCM) in the detection of antibodies against the native S-protein from SARS-CoV-2. By contrast to other immunological techniques, FCM detects antibodies directed against the native and functional S-proteins, exposed on the membrane of a transfected cell line, allowing optimal conditions for improved sensitivity and specificity^14,15^.

## Methods

### Patients

Our study was accomplished through the analysis of 50 serum samples, collected from November 5 to December 11, 2020 by venous and capillary puncture of patients infected in the first pandemic wave (between March and April, 2020). Blood samples were centrifuged for the isolation of serum/plasma. Samples were then labelled and inactivated by heat (57 °C 50′) for a safe handling. Finally, they were stored at − 80 °C until their analysis. The median time elapsed since the first qPCR + until the FCM study was 249 days (range 220–271). In addition, we included in the study 50 pre-pandemic samples (May–June 2019) as healthy negative controls. The mean age of patients and controls was 46 years (range 22–66) and sex distribution was similar between male and female.

### Cell lines

We have used a stably transfected non-adherent Jurkat cell line that expresses both the full-length native S-protein of SARS-CoV-2 and a truncated form of the human EGFR protein (S-Jurkat). These cells express simultaneously variable amounts of EGFR and S-protein, as previously described^14^. We also included a second wild type Jurkat cell line that was added to each test tube as negative internal fluorescence control (0-Jurkat).

### Sample processing

Our strategy was based on the technique described by Horndler et al^14^, with the introduction of some modifications such as the addition of a viability reagent to exclude non-viable cells from the analysis.

For each individual assay, we prepared a mixture with 50.000 0-Jurkat and 150.000 S-Jurkat cells in a single tube. The cell suspension was incubated with a 1:50 dilution of serum samples in PBS^14^ for 20 min on ice. Then, cells were washed with PBS and stained for additional 20 min with a mixture of BD Via-Probe-FITC (BD biosciences), anti-IgG-PerCP. (Jackson ImmunoResearch), anti-IgA-Alexa Fluor 647 (Jackson ImmunoResearch) and anti-EGFR-BV421 (Biolegend), that allowed for the selection of viable cells and for the detection of antibodies bound to S-protein expressed on transfected cells. After a final wash, samples were acquired in an Omnicyt flow cytometer (Cytognos SL) and analyzed using the Infinicyt 2.0 software (Cytognos SL).

The optical filters included in the cytometer configuration were: 530/30 for BL-1 detector (FITC), 695/40 for BL-3 detector (PerCP), 670/14 for RL-1 (Alexa Fluor 647) and 440/50 for VL-1 (BV421).

A minimum of 50,000 viable events, discarding doublets and debris were considered for the analysis. To evaluate the reproducibility of the assay, each patient was tested in two independent experiments (performed in venous and capillary blood samples respectively).

### Flow cytometry calibration

Flow cytometer target values were established using Rainbow beads (Cytognos SL) according to manufacturer’s instructions. The compensation matrix was created using CompBeads (BD Bioscences) for anti-EGFR and fresh blood for anti-IgG, anti-IgA and BD Via-Probe.

### Flow cytometry interpretation

IgG and/or IgA antibodies specifically bound to S-proteins were identified by comparing the median fluorescence intensity (MFI) of the S-Jurkat and the 0-Jurkat cells in each sample. Specific antibody binding was estimated by determining the MFI-ratio between transfected/non transfected Jurkat cells for the fluorescent signals corresponding to anti-IgG and anti-IgA (IgG MFI-ratio and IgA MFI-ratio respectively). To further validate the specificity for S-protein of the signals generated by IgG and IgA antibodies, we studied the correlation between the MFIs corresponding to IgG or IgA bound to transfected cells and EGFR expression (as a surrogate indicator of S- protein expression^14^) Supplementary Fig. 1.

### CLIA assay

In order to compare our strategy with conventional serological tests, we analyzed the samples with a commercial CLIA assay (MAGLUMI® SARS-CoV-2 S-RBD, Snibe diagnostic) following manufacturer’s instructions.

### Neutralization test

We performed a functional analysis for the assessment of neutralizing antibodies. We evaluated the creation of syncytia between S-Jurkat and HepG2 cells expressing ACE2, the cellular receptor of CoV-2 and ligand of S-protein, through the quantification of double positive cells as previously described^14^.

### Statistical analysis

All statistical analyses were performed using the 19.0 SPSS software. Results were expressed as medians, means, SDs and confidence intervals (CI). We used a linear regression model to correlate different levels of antibody expression. (*p* < 0.05).

### Ethics approval

This study has been performed in accordance with all the principles included in the declaration of Helsinki, it was approved by the Ethics Committee for Drug Research of the General University Hospital of Alicante (CEIm-HGUA). An informed consent was obtained from all the participants.

## Results

### MFI-ratio in pre-pandemic samples

Pre-pandemic samples showed an IgG mean fluorescent intensity (IgG-MFI) ratio of 1.18 (CI 95% 0.96–1.40) and IgA-MFI ratio of 1.12 (CI 95% 1.0–1.35). No significant linear correlation between IgG/EGFR and/or IgA/EGFR. (R^2^ < 0.25 *p* > 0.1) was found in the analysis of pre-pandemic serums.

### MFI-ratio in SARS-CoV-2 samples

**Figure 1:**
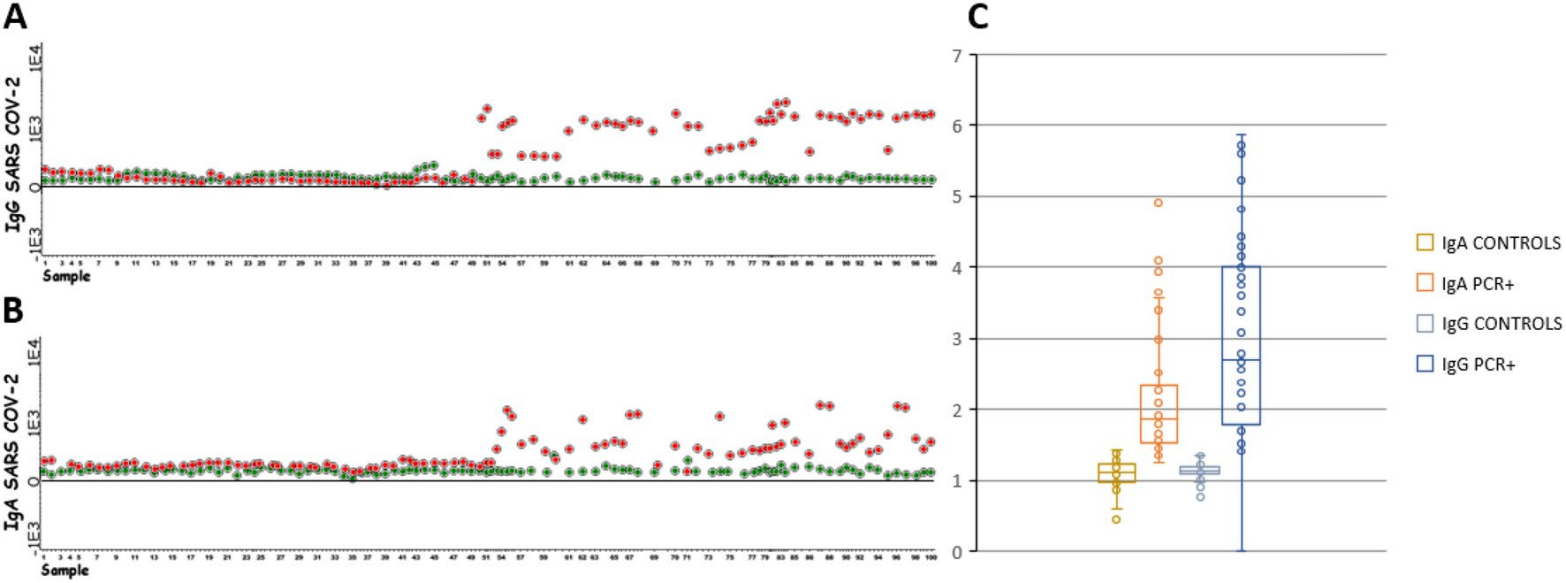
(**A**) IgG MFI of S-Jurkat (red) versus 0-Jurkat (green) cells in negative (1–50) and confirmed SARS-CoV-2 samples (51–100). (**B**) IgA MFI of S-Jurkat (red) versus 0-Jurkat (green) cells in negative (1–50) and confirmed SARS-CoV-2 samples (51–100). (**C**) MFI ratio of all analyzed samples.

**Figure 2:**
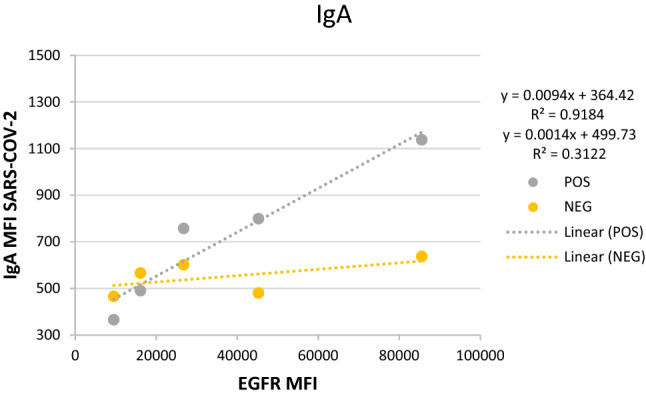
Representation of the IgA/EGFR correlation approach in a sample (grey), classified as anti-S IgG+ /IgA− by the ratio method, and a negative control (yellow). The linear regression graph shows IgA MFI versus EGFR MFI of both samples. The regression coefficient of the sample (grey) versus a negative control (yellow) demonstrate IgA seropositivity (R2 = 0.92 vs. R2 = 0.31).

All samples from PCR + patients showed an IgG-MFI ratio > 1.4 (mean = 4.12, CI 95% 1.54–7.11) and positive IgG/EGFR correlation, so they were considered anti-S/IgG+ (Fig. 1). Using the same strategy, 88% of samples were classified as anti-S/IgA+ (IgA-MFI ratio > 1.35; mean = 2.30, CI 95% 1.5–7.8 and positive anti-IgA/EGFR correlation). Nevertheless 5/6 samples initially classified as anti-S/IgG+ /IgA− using the MFI ratio method were reclassified as anti-S/IgG+ /IgA+ using the correlation IgA/EGFR method (Fig. 2). Finally, we compared samples collected by capillary puncture versus venipuncture and we could conclude that both results were identical (Supplementary Fig. 3).

### Comparison with CLIA and functional analysis

**Figure 3:**
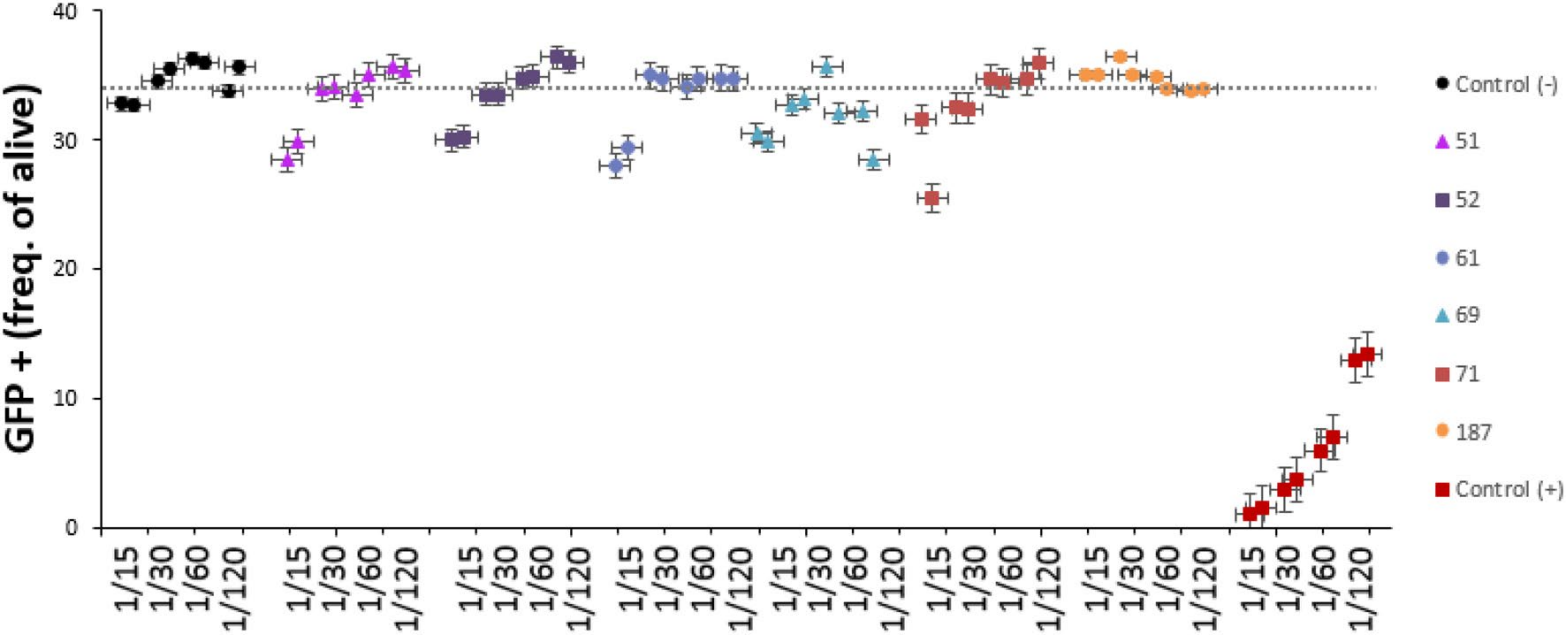
Neutralization assay in 6 dissenting samples: 5 FCM+ /CLIA− samples (51, 52, 61, 69 and 71) and 1 FCM− /CLIA+ sample (187). The reduction of S-protein lentiviral transduced ACE2-cells, due to the blockage of S-protein from serum antibodies, confirmed the presence of functional antibodies in all 5 FCM+ /CLIA− samples.

All samples were evaluated by CLIA and then compared with our FCM method. Discordant results were observed in 6 patients (5 FCM+ /CLIA− ; 1 FCM− /CLIA +). These samples were subjected to a functional assay to study the presence of neutralizing antibodies that were confirmed in all five FCM+ /CLIA− cases (Fig. 3). We could not demonstrate the presence of neutralizing antibodies in the FCM−/CLIA+ case.

## Discussion

In this study, we generated a highly sensitive FCM method to detect specific IgG and IgA antibodies against SARS-CoV-2 native S protein. Our results indicate that IgG antibodies remains detectable for at least 8 months since the first PCR+ in virtually all mild or asymptomatic patients. IgG is the most widely studied indicator of a SARS-CoV-2 infection and its detection has been reported since the first week after the infection^16^. Furthermore, previous studies performed using ELISA and/or CLIA have shown that IgG persists in up to 90% of patients for at least 6 months after acute infection^17^. Additionally, other remarkable studies have demonstrated that IgG exhibits significant neutralizing activity against S protein^18^. Nevertheless, similarly to previous studies based on FCM^13,14^ we have demonstrated that FCM can identify IgG/IgA antibodies in patients previously classified as negative by CLIA. This may reflect the increased ability of FCM methods to identify antibodies that recognize epitopes which may be present only in the native tridimensional configuration of S protein, not available in the solid phase based classic serological assays.

Most studies have focused on the diagnostic value of IgA for early diagnosis of COVID19^19^. Nevertheless, this is the first study which proves that the vast majority of mild/asymptomatic patients infected with SARS-CoV-2 develop a sustained serum IgA response persisting for months after resolution of infection. IgA not only plays a crucial role in the immunological protection of mucous membranes, but also serum IgA can inhibit the inflammatory effects of other immunoglobulins helping in the regulation of systemic immune response^20^. Increased IgA levels in the lungs have been correlated with a reduction in SARS pathology in animal models^20^. These observations have not been reproduced in humans; however, recent findings strongly suggest that human IgA contributes to the neutralization of S protein^18^. Therefore, the detection of IgA with highly sensitive techniques may become an interesting tool to define the lifespan of the protective immune response against SARS-CoV-2.

The functional assay confirmed the presence of neutralizing anti-S antibodies in all FCM+ patients, including 5 patients classified as negative by CLIA. Therefore, our strategy can be useful to identify long-lasting antibody production as well as to determine the immune status after SARS-CoV-2 infection.

Compared to CLIA/ELISA and rapid serological tests, our FCM method requires the use of a flow cytometer and cell cultures. However, the minimal amount of sample used for FCM study along with automated analysis strategies represent significative improvements in the daily routine. In addition, the use of lentiviral vectors makes possible to transfect different mutational forms of the S protein, allowing the update of the transfected cell line as needed, for the detection of immunity against new mutational variants.

In summary, the strategy presented here confirms that FCM is a highly specific and sensitive technique for the detection of antibodies against SARS-CoV-2. FCM constitute a promising tool to study long-term protective humoral immune response in cases where antibody levels were predictably low, such as in the long-term monitoring of asymptomatic patients, immunosuppressed people or elderly patients. However, additional studies would be necessary to investigate its usefulness in the routine follow-up or in the design of specific vaccination strategies.

## Supplementary Information

Supplementary Information.
