# Supplementary material for: Flow cytometry detection of sustained humoral immune response (IgG + IgA) against native spike glycoprotein in asymptomatic/mild SARS-CoV-2 infection
Source: Sci Rep. 2021 May 21;11:10716. doi: 10.1038/s41598-021-90054-4 (PMC8140089; doi:10.1038/s41598-021-90054-4)
Supplement: Supplementary file 1 — Supplementary Information. [file 41598_2021_90054_MOESM1_ESM.pdf]

Flow cytometry detection of sustained humoral immune response (IgG+IgA) against native spike glycoprotein in asymptomatic/ mild SARS-CoV-2 infection.

Paula Piñero Romero[1,*], Francisco Marco De La Calle[2], Lydia Horndler[3], Balbino Alarcón[3], Marisol Uribe Barrientos[4], Héctor Sarmiento[2], Fabián Tarín[2].

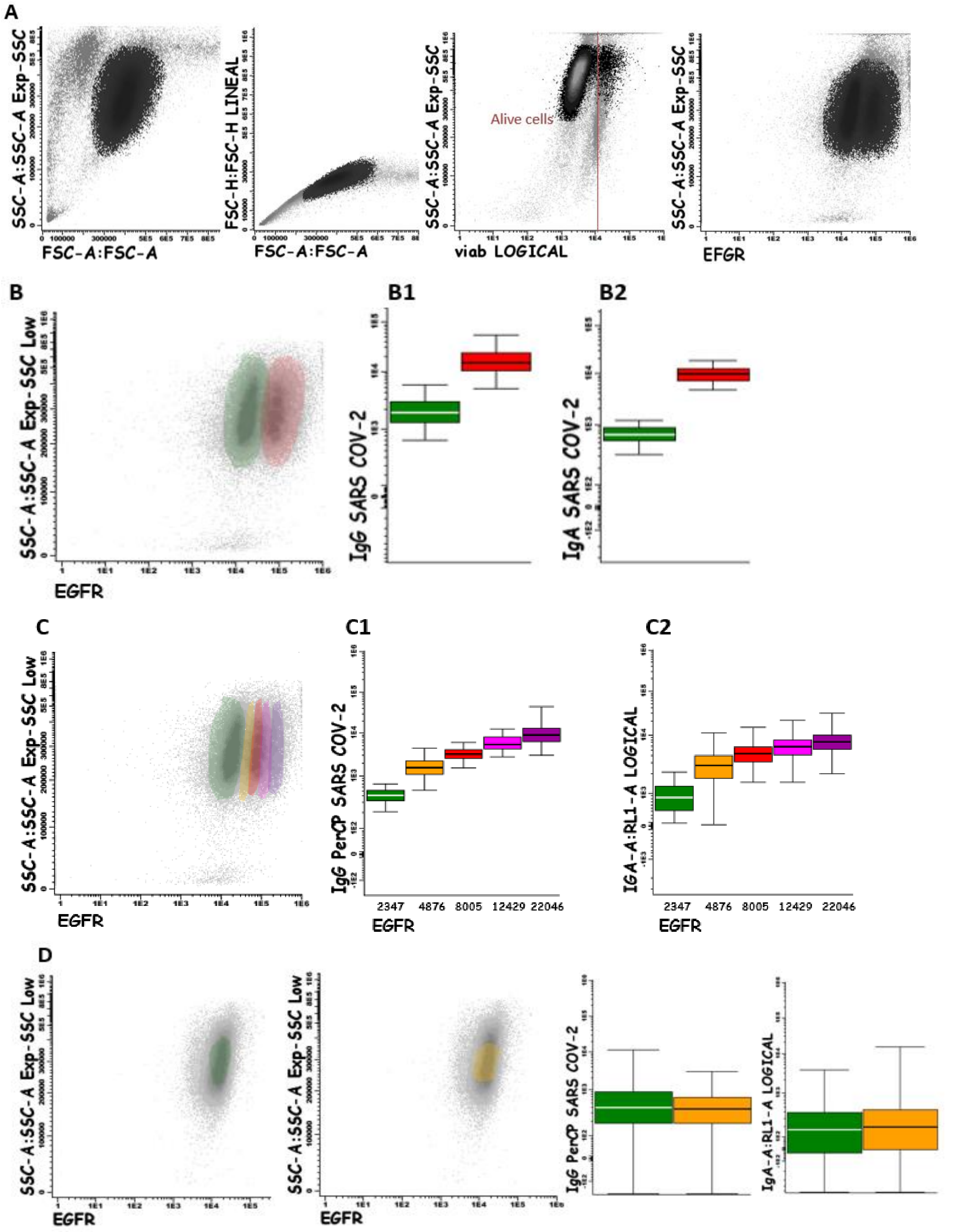

Supplementary figure 1. A) Sequential gating in the analysis of a sample. The region of analysis is represented in black and the rest of events are shown in grey. **B)** MFI of S-Jurkat cells (red)/0-Jurkat cells (green) for IgG (B1) and IgA (B2). **C)** Correlation between 5 levels (1 negative and 4 positive) of EGFR and IgG/IgA expression (C1 and C2 respectively). **D)** MFI of unstained S-Jurkat cells (yellow) and 0-Jurkat cells (green). As the image shows, there are no differences between S-Jurkat and 0-Jurkat in the unstained control.

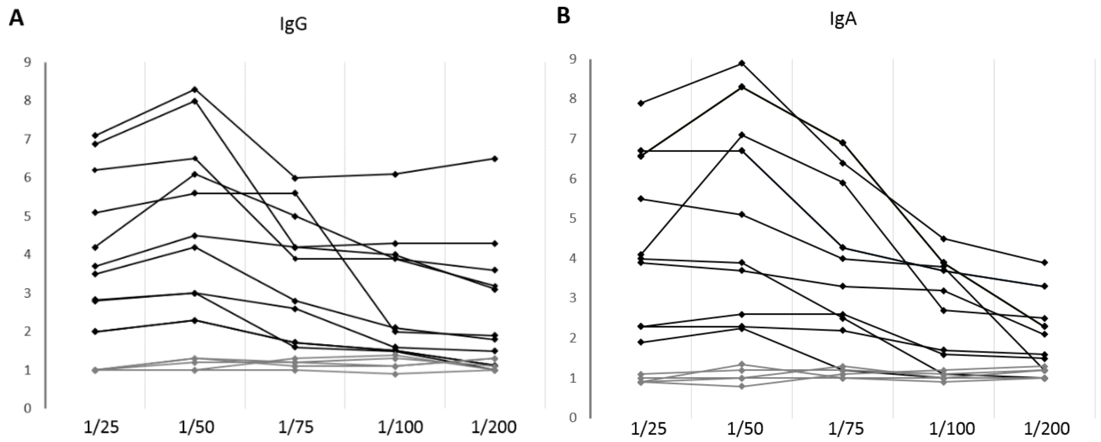

Supplementary figure 2. IgG (A) and IgA (B) ratios obtained from the titration assay of 10 positive serum samples (black) and 5 negative controls (grey).

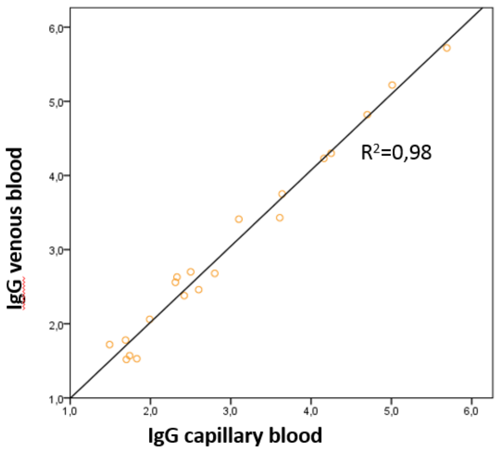

Supplementary figure 3. IgG results in a set of samples collected by venipuncture vs capillary puncture.
